# Downregulation of the CB1-Mediated Endocannabinoid Signaling Underlies D-Galactose-Induced Memory Impairment

**DOI:** 10.3389/fnmol.2020.00130

**Published:** 2020-07-28

**Authors:** Ranran Li, Zhi Huang, Juan Luo, Hongyan Luo, Wei Wang

**Affiliations:** ^1^Department of Physiology, School of Basic Medicine, Tongji Medical College, Huazhong University of Science and Technology, Wuhan, China; ^2^Department of Anesthesia, Wuhan Fourth Hospital and Puai Hospital, Tongji Medical College, Huazhong University of Science and Technology, Wuhan, China

**Keywords:** oxidative stress, endocannabinoid system, aging, learning and memory, long-term potentiation

## Abstract

Imbalance in redox homeostasis is a major cause of age-related cognitive impairment. The endocannabinoid system (ECS) is a key player in regulating synaptic transmission, plasticity and memory. Increasing evidence indicates an important interplay between the two systems. However, how excessive oxidative stress could alter ECS and that, in turn, impairs its modulatory role in synaptic plasticity and cognitive function remains elusive. In the present study, we examined this causal link in D-galactose-induced oxidative rats. First, the reactive oxygen species generating enzymes, especially nitric oxide synthase (NOS), indeed show an elevated expression in D-galactose-treated rats, and this was correlated to an impaired hippocampal long-term potentiation (LTP) and spatial memory loss in animal behavioral tests. Second, the cannabinoid receptor type I (CB1)-mediated signaling is known to regulate synaptic plasticity. We show that a decrease in CB1 and increase in degradation enzymes for CB1 ligand endocannabinoid anandamide all occurred to D-galactose-treated rats. Surprisingly, application of low-dose anandamide, known to reduce LTP under physiological condition, now acted to enhance LTP in D-galactose-treated rats, most likely resulted from the inhibition of GABAergic synapses. Furthermore, this reversal behavior of CB1-signaling could be fully simulated by a NOS inhibitor, diphenyleneiodonium. These observations suggest that interaction between redox dysfunction and ECS should contribute significantly to the impaired synaptic plasticity and memory loss in D-galactose-treated rats. Therefore, therapies focusing on the balance of these two systems may shed lights on the treatment of age-related cognitive impairment in the future.

## Introduction

Aging is generally associated with a decline of cognitive performance. Redox imbalance is a key mechanism of the aging process that is characterized by elevated reactive oxygen and nitrogen species (ROS/RNS) and/or down-regulated antioxidant abilities (Finkel and Holbrook, 2000). The disruption of redox homeostasis leads to oxidative stress that can cause direct damage to cellular architecture. The brain is particularly susceptible to the attack of oxidative stress owing to its high level of oxygen and lipid content. Aging-related cognitive impairment is largely attributed to ROS-induced oxidative damage of proteins and nucleic acids in the brain (Gemma et al., 2007).

The endocannabinoid system (ECS) has been recognized as a crucial player in the processes of learning and memory, via regulation of transmitter release, neural excitability, synaptic plasticity, and neurogenesis in brain (Katona and Freund, 2012). ECS comprises the main cannabinoid type 1 and 2 receptors (CB1 and CB2), two key endocannabinoids anandamide [*N*-arachidonoylethanolamine (AEA)] and 2-arachidonoylglycerol (2-AG), and proteins for endocannabinoid biosynthesis and deactivation (Katona and Freund, 2012). Emerging evidence suggests that the ECS activity conveys protective effects against age-related cognitive impairment. For example, a decrease in both endocannabinoids and CB1 was found in the brain tissues of older animals (Wang et al., 2003; Piyanova et al., 2015). Restoration of CB1 signaling has been shown to reverse the age-related decline in cognitive performance of old mice (Murphy et al., 2012; Bilkei-Gorzo et al., 2017).

Accumulating evidence indicates the importance of interplay between the ECS and redox-dependent processes in brain function (Kruk-Slomka et al., 2016; Paloczi et al., 2018). Indeed, activation of ECS has been shown to inhibit ROS production in neuronal cells, which is thought to underline the neuroprotective properties of cannabinoid ligands (Paloczi et al., 2018). Furthermore, changes in cellular redox homeostasis can also impact upon the function of the ECS. For example, the oxidative stress increases the expression of CB1 and CB2 (Wang et al., 2014; Ambrozewicz et al., 2018), and the biosynthesis of endocannabinoids (Matthews et al., 2016). In addition, cellular enzymes responsible for ROS production, such as NADPH oxidase, cyclooxygenase -2 (COX-2) and nitric oxide synthase (NOS), have been shown to contribute to the metabolism of ECS components (Yang et al., 2008; Lipina and Hundal, 2016; Matthews et al., 2016).

It has not been elucidated, however, the effect of oxidative stress on ECS-mediated modulation of synaptic plasticity during brain aging. Through induction of oxidative stress and inflammatory responses, the D-galactose (D-gal) has been used as an experimental aging model that generates measurable synaptic dysfunction and memory loss (Shwe et al., 2018). In the present study we used D-gal to establish ROS-induced brain aging model to explore its detrimental downstream impact on ECS, hippocampal long-term potentiation (LTP) and animal spatial memory loss *in vivo*. We show that the chronic oxidative stress is correlated with a decline in neuronal CB1 signaling, leading to an impaired hippocampal LTP and memory loss.

## Materials and Methods

### Animals

**FIGURE 1:**
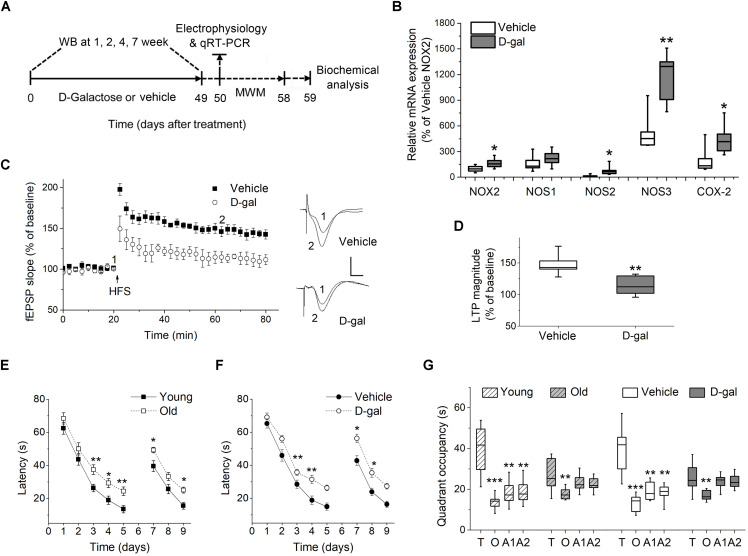
Chronic D-galactose (D-gal) treatment increased the mRNA level of several ROS-producing enzymes and induced memory deficits in rats. **(A)** Experimental setup used for assessing the behavioral and cellular responses to chronic D-gal administration. MWM, Morris water maze; WB, western blot. **(B)** A box graph showed the quantification of mRNA of interest by qRT-PCR in freshly isolated hippocampal CA1 neurons from rats received a 7-week vehicle or D-gal treatment, including NADPH oxidase isoform NOX2, neuronal nitric oxide synthase (NOS1), inducible nitric oxide synthase (NOS2), endothelial nitric oxide synthase (NOS3), and cyclooxygenase-2 (COX-2). Results were from six independent rats with triple wells. Data were normalized to the NOX2 mRNA level in control animals. **(C)** Time course of hippocampal long term potentiation (LTP) in the CA1 region after high-frequency stimulation of Schaffer collateral (HFS, arrow). *Right*, Representative fEPSP traces before (1) and 40 min after HFS (2) in vehicle and D-gal-treated animals. **(D)** Quantification of fEPSP slope change reveals a D-gal-induced occlusion of LTP (*n* = 5) when compared to the control (*n* = 6). The quantification was made by averaging normalized fEPSPs slope from 40 to 45 min after HFS. Calibration: 5 ms, 0.5 mV. *P < 0.05, **P < 0.01, compared with the vehicle group as determined by Student’s *t*-test in **(B,D)**. **(E)** Acquisition and reversal phases of the MWM in rats aged 3 months (young) and 18 months (old). **(F)** Acquisition and reversal phases of the MWM in vehicle- and D-gal-treated rats. **(G)** Probe trial phase of the MWM tested at day 6. To show quadrant preferences, the time in the target quadrant was compared to the time in the rest three quadrants in each animal group (T, target quadrant; O, opposite quadrant; A1, adjacent 1 quadrant; A2, adjacent 2 quadrant). Young, *n* = 11; Old, *n* = 10; Vehicle, *n* = 8; D-gal, *n* = 13 rats. Statistical significance was calculated using two-way repeated measures ANOVA in **(E,F)** and one-way ANOVA in **(G)**, followed by Bonferroni’s *t*-test. *P < 0.05, **P < 0.01, ***P < 0.001.

Male Sprague-Dawley (SD) rats were provided from the Laboratory Animal Center, Tongji Medical College, Huazhong University of Science and Technology (HUST). The rats were housed under a 12:12 h light/dark cycle with food and water provided *ad libitum*. Chronic systemic D-gal exposure to induce aging was performed by an established method (Cui et al., 2006). After 1-week acclimatization to the cages, the rats (5 weeks old, weighing 90 ± 5 g) were randomly divided into two groups, receiving intraperitoneal injection (i.p.) of 0.9% saline (control) or 125 mg/kg D-gal once daily for 7 weeks, respectively. The following experiments were performed at the end of the seventh week of treatment, except that the western blot was done at indicated time points and biochemical analysis was carried out when the animals finished the behavioral tests (Figure 1A). In some experiments, rats aging 3 and 18 months were employed as natural young and old controls to verify the effect of D-gal on the induction of aging. All efforts were made to minimize the suffering and number of animals used in this study.

### Morris Water Maze Test

Spatial learning and memory were evaluated in the Morris water maze (MWM) task as described (Vorhees and Williams, 2006). In the acquisition phase, the animals were given four trials daily (2 min maximum, with an interval of 1 h) for 5 consecutive days. The hidden platform remained at a fixed location for the entire acquisition period. For each trial, the latency to reach the platform was calculated. A probe trial was given at day 6, when the platform was removed. The time spent in the target quadrant was measured and compared to the time spent in the other quadrants (quadrant occupancy). To assess reversal learning, we administered another set of four trials per day between days 7 and 9, by relocating the platform to a new quadrant. Animals that did not swim or search for the goal were excluded from the analysis and further tests. The criteria were pre-established. The investigator was blinded to treatment. Recording and analysis of behavior were carried out with a video tracking system (EthoVision System; Noldus, Wageningen, Netherlands).

### Biochemical Analysis for Oxidative Stress Markers and Antioxidant Parameters

Antioxidant parameters in hippocampus were determined by measuring total antioxidant capacity (TAC), total activity of superoxide dismutase (T-SOD) and catalase activity (CAT). SOD can inactivate superoxide anion by converting it to hydrogen peroxide (H_2_O_2_). Catalase acts to neutralize H_2_O_2_ by catalyzing its conversion to water. Oxidative stress was assessed by detecting levels of ROS, H_2_O_2_ and malondialdehyde (MDA). Tissue MDA level is a marker of lipid peroxidation. All parameters were measured by commercial kits (Nanjing Jiancheng Bioengineering Institute, Nanjing, China), according to the manufacturer’s instructions. Each experiment was carried out in triplicate for each group. Hippocampus was isolated and homogenized (1/10 w/v) in cold Tris-HCl buffer (pH 7.4) for 20 s. The homogenates were centrifuged at 12,000 *g* for 15 min at 4°C. For ROS assay, samples were centrifuged at 2,400 *g* for 10 min. The supernatants were collected and the protein concentration was determined by a BCA Protein Assay Kit (Thermo Fisher Scientific, Rockford, IL, United States).

#### Determination of TAC

Total antioxidant capacity was measured with 2,2′-azino-bis-3-ethylbenzthiazoline-6-sulphonic acid (ABTS) method, using Trolox Equivalent (1 mmol/L) as the standard. Antioxidants in the sample reduce dark green colored ABTS radical to colorless ABTS form. The absorbance change at 405 nm is related to the total antioxidant level of the sample. Obtained data were expressed in units per mg of protein.

#### Determination of T-SOD Activity

The assay for T-SOD was based on its ability to inhibit the oxidation of xanthine. Xanthine–xanthine oxidase reaction system generates superoxide radicals which react with nitroblue tetrazolium to form red formazan dye. One unit of SOD activity was defined as the amount of the enzyme that reduced the absorbance at 505 nm by 50%. Enzyme specific activity was expressed in units per mg of protein.

#### Determination of CAT Activity

This method was based on the reaction of undecomposed H_2_O_2_ with ammonium molybdate to produce a yellowish color that has a maximum absorbance at 405 nm. Enzyme specific activity was expressed in units per mg of protein.

#### Determination of ROS Levels

Reactive oxygen species levels were estimated by a spectrofluorometric method using 2,7-dichlorofluorescein diacetate (DCFH-DA). Intracellular ROS reacted with the fluorogenic sensor to form a fluorescent product DCF, proportional to the amount of ROS present. The fluorescence was measured with excitation at 484 nm and emission at 530 nm. The units were expressed as AFU/mg of protein (AFU: Arbitrary fluorescence units).

#### Determination of H_2_O_2_ Concentration

It was based on the oxidation of molybdic acid by H_2_O_2_, resulting in the formation of a yellowish compound demonstrating increased absorbance at 405 nm. The results were expressed as nmol/L.

#### Determination of MDA Levels

Free MDA in hippocampal homogenates was measured by the thiobarbituric acid (TBA) reaction, via monitoring the red chromogen at 532 nm. Tetramethoxypropane was used as the standard. MDA levels were expressed as nmol per mg of protein.

### Electrophysiology *in vivo*

#### Recordings of Field Excitatory Postsynaptic Potentials (fEPSPs) in Hippocampal CA1 Area

Pentobarbital-anesthetized rats (50 mg/kg, i.p.) were positioned in a stereotaxic frame (SN-3, Narishige Instruments, Tokyo, Japan), where the skull was maintained in a flat position (the height difference between bregma and lambda was 1 mm). Rectal temperature was maintained at 37°C. A monopolar insulated tungsten wire (75 μm in diameter) was used to stimulate the Schaffer collateral afferent pathway (from bregma, in mm: AP 5.1, ML 3.8, DV 3.1 ± 0.1). fEPSPs were recorded in CA1 stratum radiatum (in mm: AP 3.5, ML 2.6, DV 2.5 ± 0.1) with a bipolar recording electrode. Test pulses (100 μs duration) were collected every 30 s by a BL420 Physiological Signals Acquisition System (Techman, Chengdu, China). The intensity was adjusted to evoke fEPSP amplitudes that were ∼50% of the maximal response. Responses in CA1 area were monitored for ≥20 min to ensure a stable baseline. Then high frequency stimulation (HFS) was applied to induce long term potentiation (LTP). Drugs or vehicles were applied by intra-hippocampus microinjection or intracerebroventricular (i.c.v.) infusion before the delivery of HFS. LTP-inducing HFS consisted of four 0.5 s trains of stimuli (200 Hz), given 2 s apart. Post-HFS responses were recorded for at least 60 min. An averaging slope of the fEPSPs over the last 10 min baseline was considered to be 100%. The following slope changes in response to drug application or HFS were expressed as a percentage of this baseline. LTP was defined as ≥20% increase in the averaging fEPSPs slope evoked between 40 and 45 min after LTP induction compared with those recorded during the 5 min immediately before LTP induction. The sites of stimulation and recording electrodes were routinely verified by postmortem examination.

#### Intra-Hippocampus Microinjection

AEA (1 μM), AM281 (100 μM, a CB1 selective antagonist) and muscimol [100 μM, a γ-aminobutyric acid (GABA) A receptor agonist] were administrated to the hippocampus before the delivery of HFS, by a stainless steel bistratal cannula. The guide cannula (0.6 mm outer diameter) was sticking tightly to the recording electrode. The infusion cannula (0.3 mm outer diameter) was connected via polyethylene tube to a Hamilton 2 μl syringe. When the infusion cannula was fully inserted through to the guide cannula, the tip of the recording electrode protruded 0.2 mm beyond that of the infusion cannula. Therefore, the infusion cannula was aimed at the pyramidal cell layer of CA1 in the dorsal hippocampus. Vehicle infusions were either saline or saline containing 1% DMSO, depending on the solvent using in drug preparation. A volume of 0.5 μl drug solution or vehicle was injected over a period of 1 min and the injection cannula was withdrawn 2 min later. At the end of each experiment, animals were microinjected with 0.5 μl of pontamine sky blue through the cannula to label the injection sites. Data from subjects in which the cannula tips were in the appropriate brain region were included in the statistical analyses.

#### I.c.v. Infusions

It was employed to deliver NADPH oxidase inhibitor acetovanillone (ACE, 100 μM), NOS inhibitor diphenyleneiodonium (DPI, 200 μM) and selective COX-2 inhibitor NS398 (250 μM). The cannula was located in the lateral cerebral ventricle (from bregma, in mm: AP 0.8, ML 1.1, DV 4.0). Injections (6 μl) were made over a 5 min period by a syringe pump and HFS was applied 35 min later.

### Western Blot Analysis

Hippocampi were homogenized in RIPA buffer containing protease inhibitors, and clarified by centrifugation (12,000 *g* for 15 min). Protein concentrations were determined using a BCA Protein Assay Kit (Thermo Fisher Scientific). Equal amounts of protein (25 μg/well) were run on a 10% SDS-PAGE gel followed by transfer to nitrocellulose membranes. After blocked with 5% non-fat milk, the blots were incubated with primary antibodies to CB1 (1: 500, Abcam, ab23703), CB2 (1: 500, Abcam, ab3561), fatty acid amide hydrolase (FAAH, 1: 500, Abcam, ab54615), monoacylglycerol lipase (MAGL, 1:800, Abcam, ab24701) or GAPDH (1:10000, Sigma-Aldrich) at 4°C overnight. After secondary antibody incubation (Bioss, Beijing, China), immunoreactivity was detected with an enhanced chemiluminescent detection (Thermo Fisher Scientific). All the proteins were detected from the same membrane by using stripping buffer to strip off the first primary antibody and re-probing with the second one. GAPDH was used as a loading control. Blots were scanned and analyzed using ImageJ software. The expression level of CB1, CB2, FAAH, and MAGL were normalized to that of GAPDH.

### Quantitative Real-Time PCR (qRT-PCR) Analysis

#### Fresh Isolation of Neurons and Astrocytes and RNA Extraction

Coronal hippocampal slices at 400 μm thickness were sectioned and transferred to oxygenated (95% O_2_/5% CO_2_) aCSF at 34°C supplemented with astrocytic marker sulforhodamine101 (SR101, 1 μM) (Nimmerjahn et al., 2004). The aCSF contained (in mM): 125 NaCl, 25 NaHCO_3_, 1.25 NaH_2_PO_4_, 3.5 KCl, 2 CaCl_2_, 1 MgCl_2_, and 10 glucose. After incubation with SR101 for 30 min, the CA1 regions were dissected out from slices and placed in aCSF containing 24 U/ml papain and 0.8 mg/ml L-cysteine for 10 min. Then slices were gently triturated into a cell suspension, and plated into a recording chamber on a motorized inverted fluorescent microscope with constant aCSF perfusion. Astrocytes were identified by their positive SR101 fluorescent staining. Typical pyramidal shaped neurons showing no SR101 staining were collected separately. In each run, thirty neurons or astrocytes were collected.

#### Quantitative Real-Time PCR (qRT-PCR)

**FIGURE 2:**
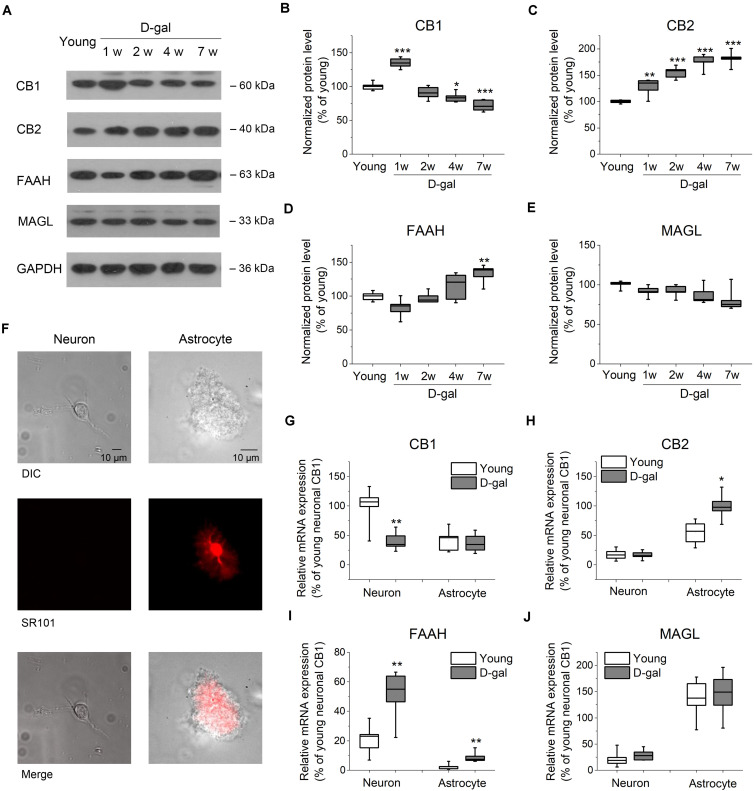
D-gal treatment altered protein and mRNA expression of key ECS components in hippocampus. **(A)** Western immunoblot analysis of the protein expression of cannabinoid receptor CB1 and CB2, fatty acid amide hydrolase (FAAH), monoacylglycerol lipase (MAGL) in hippocampus. Protein was analyzed at 1, 2, 4, and 7 weeks (w) after D-gal injection. Rats with a 7-week injection of vehicle (0.9% NaCl) were used as young control. GAPDH was used as loading control. **(B)** Quantification of CB1 protein in hippocampal tissue. The densitometric data are normalized by GAPDH and presented as a percentage of the level of young rats. **(C–E)** Similar to **(B)**, except that CB2, FAAH, and MAGL were measured in **(C–E)**, respectively. *n* = 4–5 rats/group, *P < 0.05, **P < 0.01, ***P < 0.001, compared with the young control as determined by one-way ANOVA followed by Bonferroni’s *t*-test. **(F)** Morphology of freshly isolated neuron and astrocyte from rat hippocampus CA1 area. Scale bar: 10 μm. Pyramidal neurons were selected based on their morphology and negative SR101 staining, and astrocytes were chosen based on their positive SR101 staining. These cells were harvested separately for qRT-PCR analysis. **(G)** Quantification of CB1 mRNA by qRT-PCR in freshly isolated neurons and astrocytes from rats received a 7-week vehicle (young) or D-gal treatment. **(H–J)**, Similar to **(G)**, except that mRNA of CB2, FAAH, and MAGL were measured in **(H–J)**, respectively. Results were from six independent rats with triple wells. Data were normalized to the expression level of CB1 mRNA in neurons from young animals. *P < 0.05, **P < 0.01, compared with young control in each gene group by Student’s *t*-test.

Immediately after cell harvesting, RNA extraction was done using RNeasy mini kit (Qiagen, Valencia, CA, United States). Then the RNA was converted into cDNA using Applied Biosystem’s High Capacity cDNA Reverse Transcription Kit (Thermo Fisher Scientific). The PCR primer pairs for identification of CB1, CB2, FAAH, MAGL, NADPH oxidase gene NOX2, three NOS isoforms [neuronal NOS (nNOS/NOS1), inducible NOS (iNOS/NOS2) and endothelial NOS (eNOS/NOS3)], COX-2 and GAPDH are listed in Table 1. SYBR Select Master Mix (Thermo Fisher Scientific) was used and the PCR assay was performed using a StepOnePlus PCR System (Applied Biosystems, Darmstadt, Germany). Samples were run as triplicates. GAPDH was used as the internal reference and routinely run in parallel with targeted genes. The threshold cycle (*C*t) was calculated. The expression levels of target genes were determined by 2^–ΔCT^, where ^Δ^CT was referred to the Ct difference between gene of interest and GAPDH. The mRNA level of one of the target genes was set to 100% (NOX2 of control rat in Figure 1 and CB1 in neurons from young rats in Figure 2). The individual animal’s values were normalized to it.

**TABLE 1:** Primers for quantitative Real-Time PCR analysis.

Target gene	Primer sequence	Accession number
CB1	F: 5′ GTGAACCCCATCATCTAT 3′	NM_012784.3
	R: 5′ ATCTTAACGGTGCTCTTG 3′	
CB2	F: 5′ CTTGGTGTCATGTGGGTC 3′	NM_020543.3
	R: 5′ GAGGTAGTCGTTGGGGAT 3′	
FAAH	F: 5′ TTGGGAGACCTGATCTTA 3′	NM_024132.3
	R: 5′ GAGGACGCATACTGTTGA 3′	
MAGL	F: 5′ CCTCATCTTCGTGTCCCA 3′	NM_138502.2
	R: 5′ TCCGATACCACCATCCTC 3′	
NOX2	F: 5′ GGAAACCCTCCTATGACT 3′	NM_023965.1
	R: 5′ GAAAATGTATTGTCCCACC 3′	
NOS1	F: 5′ GAGGAGGACGCTGGTGTAT 3′	NM_052799.1
	R: 5′ GGCGGTTGGTCACTTCATA 3′	
NOS2	F: 5′ GGAAAACCCAAGGTCTACG 3′	NM_012611.3
	R: 5′ CACATCGCCACAAACATAAA 3′	
NOS3	F: 5′ CACGAGGACATTTTCGGACT 3′	NM_021838.2
	R: 5′ AGGTGTTTCTTGGGTAGGC 3′	
COX-2	F: 5′ TTCCAACCCATGTCAAAAC 3′	NM_017232.3
	R: 5′ TGTCAGAAACTCAGGCGTA 3′	
GAPDH	F: 5′ GCAAGTTCAACGGCACAG 3′	NM_017008
	R: 5′ GCCAGTAGACTCCACGACAT 3′	

*F, forward; R, reverse.*

### Drugs

AEA and AM281 were purchased from Tocris (Tocris Cookson Inc., Bristol, United Kingdom). Muscimol and NS398 were from Calbiochem (La Jolla, CA, United States). All other drugs and chemicals were obtained from Sigma (St. Louis, MO, United States). D-gal and muscimol were prepared in 0.9% NaCl. AEA, AM281, ACE, DPI, and NS398 were dissolved in DMSO and diluted into working solution by 0.9% saline, with the final DMSO concentration set as 1%. Vehicle consisted of either normal saline or 1% DMSO saline solution.

### Statistical Analysis

Results were expressed as mean ± SEM. All statistical analyses were performed by using Origin 8.0 (OriginLab, Northampton, MA, United States). Comparisons between two groups were made with Student’s *t*-test. Comparisons between more than two groups were performed using either the one-way ANOVA followed by Bonferroni’s *t*-test or two-way repeated measures ANOVA. A *p*-value of <0.05 was considered statistically significant, ^∗^, ^∗∗^, and ^∗∗∗^ refer to *P*-values lower than 0.05, 0.01, and 0.001, respectively.

## Results

### Chronic D-Gal Treatment Induces Oxidative Stress and Memory Impairment in Brain

Experimental setup shown in Figure 1A was used for assessing the behavioral and cellular responses to the D-gal treatment. First we tested the effect of chronic systemic D-gal exposure on the redox balance in hippocampus, a cellular substrate for mammalian learning and memory. NADPH oxidase, NOS and COX-2 are critical enzymes responsible for cellular ROS/RNS production (Lipina and Hundal, 2016). We tested the mRNA level of NADPH oxidase gene NOX2, three NOS isoforms (nNOS, iNOS, and eNOS), and COX-2 by qRT-PCR in isolated hippocampal neurons from D-gal and vehicle treated rats. NOX2 is most likely the predominant NADPH oxidase isoform expressed in rat hippocampal neurons (Nayernia et al., 2014). As shown in Figure 1B, the level of NOX2 mRNA was increased by 64% in neurons after the D-gal application (% of vehicle NOX2: vehicle 100 ± 14.5% vs. D-gal 164.6 ± 23%, *P* = 0.03, *n* = 6). Furthermore, D-gal treatment induced a significant increase in mRNA expression of iNOS and eNOS, but not nNOS (nNOS, vehicle 161 ± 37.3% vs. D-gal 222.4 ± 35.8%, *P* = 0.26; iNOS, vehicle 15.2 ± 5.8% vs. D-gal 79.5 ± 22.5%, *P* = 0.02; eNOS, vehicle 524.1 ± 89.1% vs. D-gal 1187.8 ± 116.7%, *P* = 0.02; all *n* = 6; Figure 1B). The mRNA level of COX-2 also was significantly elevated by D-gal treatment (vehicle 197.6 ± 62.5% vs. D-gal 444.3 ± 74.4%, *P* = 0.03, *n* = 6; Figure 1B). Allied with the enhancement of ROS-producing enzymes, D-gal-treated rats were associated with significant increases in the levels of ROS, H_2_O_2_ and MDA, indicating a high level of oxidative stress and lipid peroxidation (Table 2). On the contrary, total antioxidant capacity and the activity of protein antioxidants (total SOD and CAT) were significantly decreased in D-gal-treated animals (Table 2). The results support that D-gal treatment induced redox imbalance in brain, consisting with the previous reports (Rehman et al., 2017; Shwe et al., 2018).

**TABLE 2:** The levels of oxidant and antioxidant parameters in hippocampal tissues.

Analyzed parameters	Young	Old	Vehicle	D-gal
TAC [U/mg protein]	2.011 ± 0.111	1.531 ± 0.118^a^	1.888 ± 0.140	1.350 ± 0.092^a,c^
Total SOD [U/mg protein]	1.648 ± 0.106	1.377 ± 0.115	1.619 ± 0.138	1.178 ± 0.077^a,c^
CAT [U/mg protein]	7.631 ± 0.630	6.29 ± 0.64	6.674 ± 0.612	4.116 ± 0.307^a,b,c^
ROS [AFU/mg protein]	100 ± 7.84	172.37 ± 14.8^a^	107.82 ± 11.48	204.61 ± 17.01^a,c^
H_2_O_2_ [nmol/L]	55.626 ± 7.318	75.893 ± 8.521	61.907 ± 9.668	97.737 ± 7.548^a,c^
MDA [nmol/mg protein]	0.619 ± 0.066	1.233 ± 0.073^a^	0.746 ± 0.106	1.507 ± 0.146^a,c^

*Hippocampal tissues were from rats aged 3 months (young), 18 months (old), and vehicle- or D-gal-treated rats. n = 8–11 rats/group (for ROS assay, n = 5–6 rats/group). ^a^P < 0.05 when compared with young group. ^b^P < 0.05 when compared with old animals. ^c^P < 0.05 when compared with vehicle-treated control group; one-way ANOVA with Bonferroni’s t-test.*

Next, we investigated the effect of D-gal on the induction of hippocampal LTP and memory performance. D-gal treatment impaired LTP by a ∼40% inhibition in the Schaffer collateral-CA1 pathway compared with vehicle controls (vehicle, 147.2 ± 6.7%, *n* = 6 vs. D-gal, 114.3 ± 7.3%, *n* = 5, *P* = 0.009; Figures 1C,D). Spatial learning and memory in rats were analyzed using Morris water maze (MWM) test starting on day 50 after a 7-week application of D-gal or vehicle (Figure 1A). Rats aging 3 and 18 months (termed young and old, respectively) were employed as natural status controls. The old and D-gal-treated animals learned the task more slowly than the young and vehicle-treated animals in the acquisition period (days 1–5) and the reversal phase (days 7–9), evidenced by long escape latencies (Figures 1E,F). In the probe trial phase of the test (day 6), the old and D-gal-treated animals showed impairments of long-term spatial memory, as indicated by reduced time spent in the target quadrant while controls (both young and vehicle-treated rats) displayed significant target quadrant preference (Figure 1G). There was no difference between the old and D-gal-treated rats in their learning and memory performance. Together, these results support chronic D-gal treatment can induce redox imbalance and hippocampus-dependent memory deficits that mimics natural brain aging, in good agreement with previous reports (Rehman et al., 2017).

### D-Gal Treatment Alters the Expression Level of Key ECS Components in Hippocampus

It has been demonstrated that a lower level of endocannabinoids and CB1 receptor were found in the brain tissues of older animals (Wang et al., 2003; Piyanova et al., 2015). To investigate whether the expression level of ECS was altered in D-gal-treated hippocampus, we tested the protein expression of the main ECS components, including CB1, CB2, and two key enzymes, FAAH and MAGL. To distinguish from the rats treated with the vehicle microinjection in brain, vehicle (0.9% NaCl, i.p.)-treated rats were termed as young group in the following experiments, since they had same age (3 months old at the end of the 7-week injection), memory performance and redox status as young animals shown in Figure 1. Western blot analysis revealed a significant increase of CB1 protein level in hippocampus tissues at the early stage of D-gal treatment (week 1; young, 100 ± 2.3%, *n* = 5 vs. D-gal, 134.8 ± 3.5%, *n* = 5, *P* < 0.001; Figures 2A,B). This is in line with the idea that the activation of CB1 receptors protects neurons from insults of oxidative stress and concomitant neuroinflammation (Paloczi et al., 2018). Interestingly, CB1 protein level declined from week 2 and was reduced by 28% at week 7 as compared to the young control (*n* = 5, *P* < 0.001; Figures 2A,B). As to CB2 receptor, the protein level was kept rising and showed a significant increase since the first week of D-gal treatment (Figures 2A,C). This is consistent with the observation that the activation of CB2 plays an important role in anti-inflammation process in brain (Braun et al., 2018). In the meantime, we monitored the levels of FAAH and MAGL that hydrolyze AEA and 2-AG, respectively. While the expression of MAGL was not affected by D-gal treatment (Figures 2A,E), FAAH level was elevated from week 2 and presented a significant enhancement at week 7 (young, 100 ± 1.9%, *n* = 5 vs. D-gal, 132.7 ± 6.2%, *n* = 5, *P* = 0.003; Figures 2A,D). This elevated FAAH may facilitate hydrolysis of AEA, leading to a decrease of AEA level in hippocampus.

It has been reported that cannabinoid receptors, FAAH and MAGL are expressed in both neurons and astrocytes (Benito et al., 2007; Bilkei-Gorzo, 2012; Navarrete et al., 2018). To evaluate which cell type contributes to the change of the abovementioned proteins, we next tested their mRNA levels from freshly isolated pyramidal neurons and astrocytes obtained from hippocampal CA1 area (Figure 2F). qPCR analysis revealed CB1 mRNA was distributed mainly in neurons with an albeit minor level in astrocytes. Interestingly, CB1 mRNA expression was considerably reduced in neurons after the 7-week D-gal treatment, while no significant change was observed in astrocytes (Figure 2G). The result indicated D-gal induced a CB1 downregulation mainly in neurons of hippocampus. Conversely, CB2 mRNA was mainly expressed in astrocytes and D-gal induced elevation of CB2 mRNA also only occurred in astrocytes (Figure 2H). With regards to FAAH gene, it located predominately in neurons, whereas D-gal treatment significantly enhanced the FAAH mRNA level in both neurons and astrocytes (Figure 2I). MAGL gene also was distributed in a cell specific manner, with a high expression in astrocytes. However, MAGL mRNA expression was not affected by D-gal treatment in both cell types (Figure 2J), concomitant with the protein expression. Together, these results indicate that the neuronal CB1 receptor declines in D-gal-treated hippocampus, companied with an increase of neuronal FAAH.

### CB1 Activation Facilitates Hippocampal LTP in D-Gal-Treated Rats but Impairs LTP in the Young Control

**FIGURE 3:**
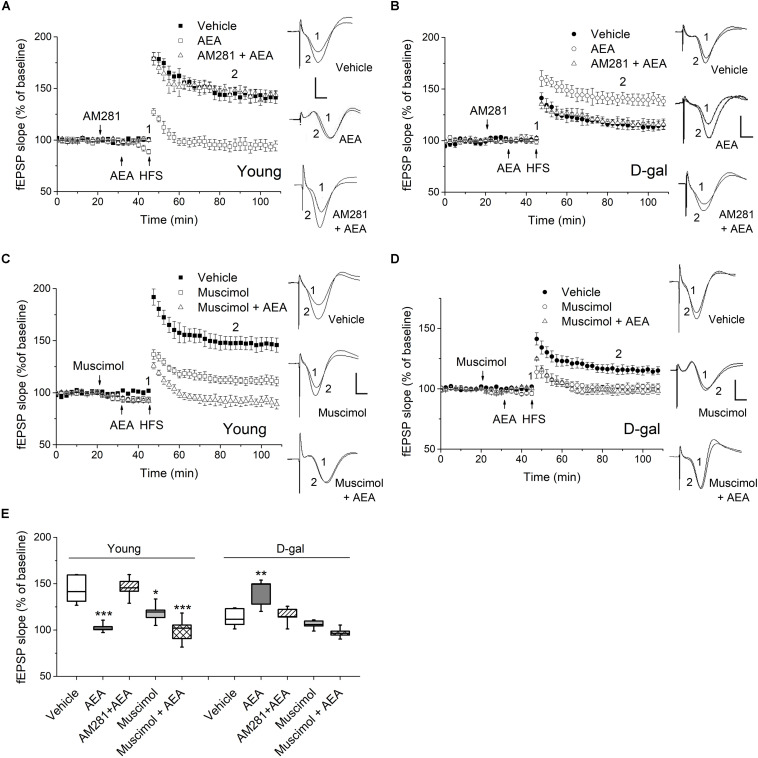
CB1 activation facilitated hippocampal LTP in D-gal-treated rats but impaired LTP in young controls. **(A)** Time course of LTP with endocannabinoid anandamide (AEA, 1 μM) or vehicle (1% DMSO) applied for 15 min prior to HFS in young rats, or with a 10 min-pretreatment of AM281 (100 μM) followed by the AEA application. Drugs were delivered via intra-CA1 injections. *n* = 5–6 rats/group *Right*, Representative fEPSP traces before (1) and 40 min after HFS (2) in young and D-gal-treated animals. **(B)** Similar to **(A)**, except that D-gal-treated rats was used for this study. Five rats were used in each group. **(C)** Time course of LTP with a 25 min intra-CA1 injection of 100 μM muscimol, a selective GABA-A receptor agonist, or vehicle (1% DMSO) prior to HFS in young rats, or with a 10 min pretreatment of muscimol followed by a 15 min AEA application. *n* = 5 rats/group. **(D)** Similar to **C**, except that D-gal-treated rats was used for this study. *n* = 5 rats/group. **(E)** Quantification of fEPSP slope change showing AEA reduced LTP in young animals but increased LTP in D-gal-treated rats, which was blocked by CB1 antagonist AM281. Pretreatment of muscimol abolished the AEA-induced LTP enhancement in D-gal treated rats. *P < 0.05, **P < 0.01, ***P < 0.001 compared with the vehicle control as determined by one-way ANOVA followed by Bonferroni’s *t*-test. Calibration: 5 ms, 0.5 mV.

There is rising acknowledgment about the interplay between ROS and endocannabinoid signaling (Kruk-Slomka et al., 2016; Lipina and Hundal, 2016). To determine whether oxidative stress alters ECS-mediated modulation of synaptic plasticity, we examined the effect of endocannabinoid AEA on the induction of hippocampal LTP in D-gal-treated rats. Interestingly, application of low-dose AEA (1 μM) inhibited LTP in young animals (vehicle, 143.37 ± 6.12%, *n* = 6 vs. AEA, 102.5 ± 2.3%, *n* = 5, *P* < 0.001; Figures 3A,E), but elevated LTP in D-gal-treated rats (vehicle, 113.2 ± 4.5%, *n* = 5 vs. AEA, 140.2 ± 6.8%, *n* = 5, *P* = 0.011; Figures 3B,E), when applied intrahippocampally 15 min prior to HFS. We also noticed that application of AEA (1 μM, 15 min) slightly reduced the baseline slope of fEPSP by 9.9 ± 0.8% (*n* = 4) in young animals, where there was no alteration in D-gal group. This reduction of baseline fEPSP may shift the threshold for LTP, thereby limiting LTP induction in young rats. Next, a selective CB1 receptor antagonist AM281 was used to determine whether the act of AEA on LTP was mediated by CB1 receptor. AM281 (100 μM) pretreatment reversed the AEA effect on LTP in both young and D-gal-treated rats (Figures 3A,B,E). The result indicates AEA-induced alteration of hippocampal LTP is mediated via CB1 receptor pathway.

CB1 are mostly distributed presynaptically, with a highest density in GABAergic interneurons in the hippocampus. Activation of CB1 in presynaptic GABAergic terminals results in reduced GABA release (Katona and Freund, 2012). Furthermore, it has been reported chronic D-gal treatment induced a significant decrease of glutamate but no change in GABA in hippocampus (Lin et al., 2014). Therefore, we next investigated whether the GABAergic activity was involved in the diverse act of AEA on hippocampal LTP. As a selective GABA-A agonist, muscimol (100 μM) applied alone inhibited the hippocampal LTP in both young and D-gal treated rats (Figures 3C–E). However, it exerted different effects on AEA-modulated LTP. As Figures 3C,E shown, muscimol pretreatment showed no effect on the AEA-induced LTP impairment in young animals (in young rats, AEA, 102.5 ± 2.3%, *n* = 5 vs. Muscimol + AEA, 99.6 ± 6.3%, *n* = 5, *P* > 0.05). Interestingly, pretreatment of muscimol completely abolished the AEA-induced LTP enhancement in D-gal treated rats (in D-gal aging rats, AEA, 140.2 ± 6.8%, *n* = 5 vs. Muscimol + AEA, 97.1 ± 2.5%, *n* = 5, *P* < 0.001; Figures 3D,E). Thus, it supports the idea that reduced CB1-signaling fails to counteract the increased GABAnergic activity by regulating GABA release retrogradely, likely contributing to D-gal- mediated inhibition of hippocampal LTP. Taken together, these results indicates that application of low-dose AEA, known to reduce LTP under physiological condition, now acted to enhance LTP in D-gal-treated rats via a GABA-A receptor-mediated mechanism.

### D-Gal-Induced Elevation of NOS Activity Is Correlated to the Impaired Modulatory Role of AEA in Hippocampal LTP

**FIGURE 4:**
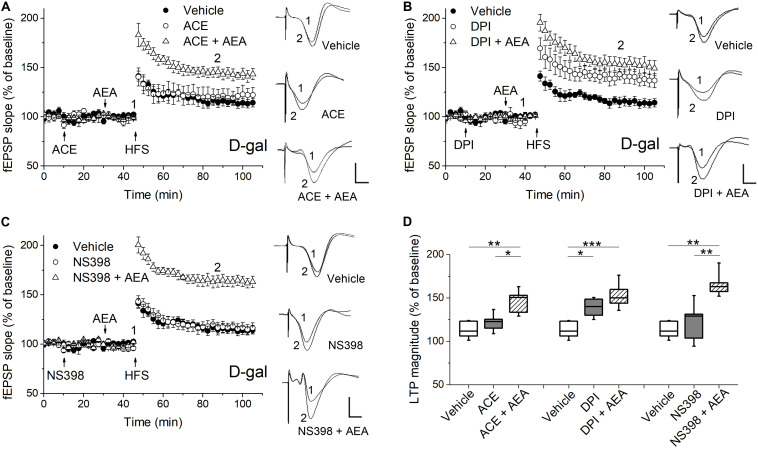
Pharmacological blockade of the ROS-producing enzymes affected AEA-modulated LTP in hippocampus of D-gal-treated rats. **(A)** Time course of LTP with a 35 min infusion of the NADPH oxidase inhibitor acetovanillone (ACE, 100 μM, i.c.v), or a 20 min ACE infusion followed by a 15 min AEA introhippocampal application (1 μM), or only vehicle treatment prior to HFS in D-gal-treated rats. *Right*, representative fEPSP traces before (1) and 40 min (2) after HFS. **(B,C)** Similar to **(A)**, except that a non-selective NOS inhibitor diphenyleneiodonium (DPI, 200 μM, in **B**) and a selective COX-2 inhibitor NS398 (250 μM, in **C**) were used for the study, respectively. The vehicle was normal saline solution containing 1% DMSO. It was used in both i.c.v infusions and intrahippocampus microinjections. The fEPSP data in vehicle groups in **(A–C)** were from the same five rats. **(D)** Quantification of fEPSP slope change indicated the ROS-producing enzymes conveyed different effects on AEA-modulated LTP. All *n* = 5. *P < 0.05, **P < 0.01, ***P < 0.001 as determined by one-way ANOVA followed by Bonferroni’s *t*-test. Calibration: 5 ms, 0.5 mV.

It has been reported that the ECS signaling are modulated by several critical enzymes responsible for cellular ROS/RNS production, such as NADPH oxidase, NOS and COX-2 (Lipina and Hundal, 2016). Furthermore, we have observed D-gal-treatment significantly increased the mRNA level of NADPH oxidase gene NOX2, iNOS, eNOS, and COX-2 in hippocampal neurons (Figure 1B). To determine whether the ROS generating enzymes functionally affected AEA-modulated synaptic plasticity, we tested the hippocampal LTP by pharmacologically blocking the abovementioned enzymes. First, we investigated the effect of NADPH oxidase on the LTP induction. The activation of NADPH oxidase has been shown to enhance 2-AG level and CB1 expression in non-neuronal cells (Wang et al., 2014; Matthews et al., 2016). As shown in Figures 4A,D, the application of ACE (100 μM, i.c.v.), a cell-permeable selective inhibitor of NADPH oxidase, caused no change in LTP magnitude and could not reverse the act of AEA (1 μM) on hippocampal LTP in D-gal-treated rats. It indicates NADPH oxidase activation may not be involved in D-gal-induced memory deficit or ECS signaling. Next, we examined the effect of NOS inhibition on LTP induction by using DPI (200 μM, i.c.v.), which displays potent inhibition of NOS with selectivity for eNOS and iNOS over nNOS. NOS is known to produce a remarkable amount of NO in various cell types and excessive NO can contribute to the generation of peroxynitrite (ONOO^–^), a powerful oxidant and nitrating species (Matthews and Ross, 2015; Paloczi et al., 2018). D-gal-induced decrease in LTP was restored to the level of young control by the DPI application (young 143.4 ± 6.1%, *n* = 6 vs. D-gal 138.8 ± 5%, *n* = 5, *P* > 0.05). Interestingly, addition of AEA (1 μM) in the presence of DPI did not further elevate the amplitude of LTP, indicating a shared mechanism of action (Figures 4B,D). COX-2, an inducible enzyme, has been demonstrated to oxygenate AEA and 2-AG to form certain prostaglandins (Yang et al., 2008; Paloczi et al., 2018). We tested the effect of COX-2 blockage on hippocampal LTP by injecting NS398 (250 μM, i.c.v.), a selective COX-2 inhibitor. NS398 failed to affect LTP in D-gal-treated rats and it also could not reverse the AEA (1 μM) effect on hippocampal LTP (Figures 4C,D). It is noteworthy that COX-2 mRNA level was significantly elevated by D-gal treatment, indicating an increased degradation level of endocannabinoids. It may further contribute to the decline of ECS signaling in D-gal-treated brain. These results indicate that an increase of NOS activity is involved in the LTP inhibition relevant to a decline of CB1 signaling.

## Discussion

In the present study, we investigated the redox-mediated regulation of ECS activity and its role in D-gal-induced memory impairment. The results indicate the following. First, the CB1-mediated endocannabinoid signaling declined in oxidative stress-related brain aging, including a massive disruption of ECS from cannabinoids biochemical synthesis/degradation to CB1/CB2 expression. Second, the classic retrograde inhibition of CB1-signaling to LTP was reversed to be a potentiation role to LTP in D-gal-treated rats, likely resulted from an endocannabinoid-mediated reduction of GABAnergic activity. Third, this dysfunctional role of CB1-signaling in D-gal-treated rats could be recapitulated by inhibition of excessive NOS activity. Altogether, these observations suggest that interaction between oxidative stress and ECS may contribute significantly to the memory loss in D-gal-treated rats.

### The CB1-Mediated ECS Signaling Declines in Age-Related Cognitive Impairment

The ECS is one of the main neuromodulatory systems controlling over the cognition function of brain. It predominantly functions by modulating neural excitability and endocannabinoid-dependent synaptic plasticity, primarily through the well-documented retrograde activation of presynaptic CB1 receptors (Katona and Freund, 2012). Recent reports have suggested a protective role for the ECS against age-related cognitive decline. For example, mice lacking CB1 showed an accelerated age-dependent memory impairment, accompanied by a loss of principal neurons in the hippocampus and enhanced neuroinflammation (Bilkei-Gorzo et al., 2005; Albayram et al., 2011). Further evidence demonstrated that activation of CB1 by a chronic treatment of low dose of Δ9-tetrahydrocannabinol restored cognitive function in old mice, by upregulating genes with anti-aging and memory-promoting effects (Bilkei-Gorzo et al., 2017). Allied to this, in the present study, we have observed AEA (1 μM) applied locally restored the inhibited hippocampal LTP in a CB1 dependent manner in D-gal induced aging rats (Figure 3). The result supports a direct link between CB1-mediated ECS signaling and memory rescue in aging. Furthermore, a reduced expression of CB1 accompanied with an increased level of AEA degrading enzymes (FAAH and COX-2) was found in hippocampal neurons in D-gal-induced aged rats (Figures 1, 2). It is consistent with the previous study that a lower level of CB1 was present in rat hippocampus during aging (Canas et al., 2009). Our results suggest both endocannabinoid AEA and CB1 receptor are down-regulated after a chronic oxidative stress, resulting in the decrease of ECS-mediated hippocampal LTP.

Moreover, we have observed a dual act of CB1 activation on LTP induction:CB1 agonist facilitates LTP in D-gal-induced aging brain but impairs LTP in young brain (Figure 3). It is consistent with the study which showed cannabinoid treatment worsened memory performance in young mice but restored cognitive deficits in old animals (Bilkei-Gorzo et al., 2017). It has been demonstrated that endocannabinoid can impair hippocampal LTP through presynaptic inhibition of neurotransmitters essential for memory and learning processes (Katona and Freund, 2012; Basavarajappa et al., 2014). However, the majority of CB1 receptors accumulates presynaptically on GABAergic neurons in the hippocampus (Katona and Freund, 2012). Endocannabinoid-mediated inhibition of presynaptic GABA release was shown to facilitate the induction of LTP in the hippocampus, most likely by increasing neurons excitability associated with LTP (Chevaleyre and Castillo, 2004; Jiang et al., 2010). Furthermore, several studies have revealed a decrease in glutamate levels in hippocampus of D-gal model or natural aging rodents with no significant changes in GABA levels (Lin et al., 2014; Rozycka et al., 2019). These observations support the idea that excitatory/inhibitory balance is weakened in the hippocampus and shifted to the inhibitory side in old animals. In addition, GABA-A receptors are generally well preserved in the hippocampus of aged mice (Palpagama et al., 2019). Thus, it is plausible to interpret that AEA rescues the impaired LTP by inhibiting GABAergic synapses in D-gal aging brain. Indeed, it is further supported by the observation that the application of GABA-A agonist abolished the effect of AEA on hippocampal LTP in D-gal treated rats (Figure 3). Considering that ECS exhibits varied distribution patterns and multiple functions in the brain, further investigations are needed to reveal how endocannabinoid signaling at different synapses contributes to distinct cognitive components.

### Interactions Between the Oxidative Stress and ECS in Brain

D-gal treatment generates a progressive oxidative stress and neuroinflammation in brain (Shwe et al., 2018). Consistent with this, our results revealed enhanced levels of ROS and ROS producing enzymes but a decrease level of antioxidants in hippocampal tissue (Table 2 and Figure 1). An oxidative burst is known to perturb the metabolism of membrane lipids. For example, in inflammatory granulocytes of psoriatic patients, the redox imbalance was associated with decreased levels of phospholipids but with enhanced activity of enzymes involved in lipid metabolism (phospholipase A2, COX-2, etc.) (Ambrozewicz et al., 2018). Given the fact that the biosynthesis of AEA and 2-AG are mainly related to phospholipid hydrolysis (Paloczi et al., 2018), redox impairment is thought to increase endocannabinoids production. Indeed, it has been observed that the oxidative stress increases AEA and 2-AG levels and cannabinoid receptor expression (Wei et al., 2009; Ambrozewicz et al., 2018). In accord with this, we also observed there was a short-lasting elevation of CB1 and downregulation of FAAH at the early stage of D-gal treatment (Figure 2). In addition, CB2 expression level displayed a prominent increase in hippocampal astrocytes after D-gal treatment (Figure 2). Extensive work has been carried out to investigate the neuroprotective properties of ECS. It has been reported that CB1 agonists suppress intracellular ROS formation (Kim et al., 2005; Aguilera-Portillo et al., 2019) and lipids peroxidation level to alleviate neuroinflammation (Zoppi et al., 2011; Kruk-Slomka et al., 2016). CB2 activation has also been reported to attenuate oxidative stress damage and protect against neuroinflammation in several neurodisorders with cognition impairment (Javed et al., 2016; Lipina and Hundal, 2016). Therefore, the upregulation of cannabinoid receptor expression observed in the present study may be a compensatory change to ameliorate the damaging effects of ROS exposure.

However, CB1 expression subsequently declined after the 7-week D-gal treatment (Figure 2). In addition, two important enzymes responsible for endocannabinoid degradation, FAAH and COX-2, were found to be increased in extractions of hippocampus after the D-gal treatment (Figures 1, 2). FAAH mediates the degradation of AEA and COX-2 oxidative metabolism is an important pathway in degrading both AEA and 2-AG (Alhouayek and Muccioli, 2014; Paloczi et al., 2018). Therefore, our study suggests the chronic oxidative stress may reduce the level of CB1 receptor and endocannabinoids in brain. These data support the interpretation that reducing ECS activity attenuates the beneficial effects of ECS against age-related cognitive deficits, such as antioxidant, anti-inflammation and upregulation of genes involved in the memory-promoting activity (Bilkei-Gorzo et al., 2017; Paloczi et al., 2018). The question of whether AEA or 2-AG is responsible for the anti-aging activity of CB1 receptor is not known. Further investigation is needed to determine the concentration of AEA and 2-AG in hippocampus during D-gal treatment.

### The Involvement of ROS-Producing Enzymes in the ECS-Mediated Modulation of Synaptic Plasticity

Increasing number of evidences emphasize cellular ROS-generating enzymes are crucial players in the interplay between the ROS and ECS signaling systems, especially NADPH oxidase, COX-2 and NOS (Lipina and Hundal, 2016; Paloczi et al., 2018). In the present study, AEA-induced LTP restoration could be fully simulated by a NOS inhibitor in D-gal aging rats (Figure 4). NO and endocannabinoids act as retrograde messengers in synaptic plasticity (Steinert et al., 2010). Several studies indicate that a decrease in ECS signaling can facilitate NO signaling, probably by decreasing the inhibitory effects of endocannabinoid on NOS activation (Kim et al., 2006; Lisboa et al., 2015). In D-gal-induced aged brain, NO signaling would be further enhanced by the increased eNOS and iNOS expression observed in hippocampal neurons (Figure 1). It has been reported nanomolar concentrations of NO are generated by eNOS and nNOS, whereas iNOS can produce micromolar levels in response to proinflammatory stimuli (Steinert et al., 2010). Therefore, NO spillover from NOS over activation provides potential pathological sources of NO, which may lead to an impairment LTP and memory loss (Anaeigoudari et al., 2016; Wang and Han, 2018). One possible mechanism is that excessive NO can augment GABA release, regulating the strength of synaptic inputs onto hippocampal CA1 neuron (Neitz et al., 2015; Bradley and Steinert, 2016). In good agreement with it, we have observed that an increased tone of GABAergic activity might be involved in D-gal-induced LTP impairment (Figure 3). It supports the idea that reduced ECS signaling induces an abnormal NO signaling, contributing to the LTP impairment in the aged brain.

The NADPH oxidase is also a key generator of cellular ROS and its activation has been shown to increase 2-AG biosynthesis and CB1 expression in non-neuronal cells (Wang et al., 2014; Matthews et al., 2016). COX-2, one of the degrading enzymes of endocannabinoids, has been reported to increase markedly in hippocampus during normal aging (Lee et al., 2010). Indeed, we have observed that the mRNA level of NOX2 (a predominant NADPH oxidase isoform in brain) and COX-2 were increased in hippocampal neurons after the chronic D-gal application (Figure 1). However, the pharmacological block of NADPH oxidase or COX-2 caused no change in LTP magnitude and could not affect the AEA action on hippocampal LTP (Figure 4). It indicates that NADPH oxidase and COX-2 contribute little to the ECS impairment-induced LTP inhibition in D-gal-treated rats. It is noteworthy that COX-2 signaling exerts diverse effects on synaptic transmission and plasticity, depending on cell stimulus and down-stream metabolites. For example, it has been reported COX-2 mediates the antidepressant-impaired hippocampal LTP (Stachowicz et al., 2020). Nevertheless, prostaglandins derived from the endocannabinoid degradation by COX-2 have been shown to elevate hippocampal LTP (Yang et al., 2008). Further studies are required to clear how COX-2 signaling impacts on the ECS-modulated synaptic plasticity.

## Conclusion

The data presented here demonstrates the chronic oxidative stress weakens CB1-mediated ECS activity during brain aging, which leads to the impaired hippocampal LTP and memory loss. It points to a possible upregulation of the NO signaling in situations. All together, these results advance our understanding of how ECS signaling is affected by ROS in synaptic plasticity and suggest a potential therapeutic role for NOS inhibitors and modulators of the ECS in age-related cognitive impairments.

## Data Availability Statement

All datasets generated for this study are included in the article.

## Ethics Statement

All the protocols were done in accordance with the National Institute of Health Guide for the Care and Use of Laboratory Animals (NIH Publications No. 80-23) revised 1996 and were reviewed and approved by the Institutional Animal Ethics Committee of Huazhong University of Science and Technology.

## Author Contributions

RL and WW conceived the project and wrote the manuscript. RL, ZH, JL, HL, and WW conducted or assisted the research. WW supervised the project. All authors contributed to the article and approved the submitted version.

## Conflict of Interest

The authors declare that the research was conducted in the absence of any commercial or financial relationships that could be construed as a potential conflict of interest.
